# Further "Times" Correspondence

**Published:** 1920-02-28

**Authors:** Robert J. Bland

**Affiliations:** Secretary Superintendent. Royal London Ophthalmic Hospital, E.C.


### THE UNIFORM SYSTEM OF ACCOUNTS.

### Further "Times" Correspondence.

The following letters have appeared in *The Times* of February 19 and 20 respectively. The letters to which they refer were published in our last week's issue, p. 495 :—

### HOSPITAL ACCOUNTS.

*To the Editor of* The Times.

Sir,—The clear statement given by Mr. J. Danvers Power shows that the original uniform system of hospital accounts was designed by Sir Henry Burdett. But that uniform system consisted only of an income and expenditure account. It was not until Mr. J. G. Griffiths's professional help was sought in 1906 that London hospitals adopted the custom of printing balance-sheets and statistical tables in their annual reports. In connection with the history of the evolution of hospital accounts, it may be of interest to point out that, with the help of Mr. J. G. Griffiths and Sir Woodburn Kirby, the committee of the Royal London Ophthalmic Hospital in 1901 published a balance-sheet, December 31, 1900. I believe that this was the first balance-sheet published by a London hospital in its annual report.—Your obedient servant,

Robert J. Bland, Secretary Superintendent.

Royal London Ophthalmic Hospital, E.C.,
February 17.

